# The role of advanced practice nurses in improving healthcare outcomes for patients with chronic kidney disease: A scoping review protocol

**DOI:** 10.1371/journal.pone.0301676

**Published:** 2024-04-04

**Authors:** Hanako Nozu, Haruka Tamura, Takemi Kudo, Tomoko Araki, Hidetaka Sato, Takao Watanabe, Isoji Sasagawa

**Affiliations:** 1 Department of Nursing, Yamagata Tokushukai Hospital, Yamagata, Yamagata, Japan; 2 Graduate School of Medicine, Nagoya University, Nagoya, Aichi, Japan; 3 Graduate School of Health and Environment Sciences, Tohoku Bunka Gakuen University, Sendai, Miyagi, Japan; 4 Department of Urology, Yamagata Tokushukai Hospital, Yamagata, Yamagata, Japan; University of Saskatchewan, CANADA

## Abstract

**Introduction:**

The number of patients with chronic kidney disease is increasing worldwide; previous studies have suggested that advanced practice nurses, including nurse practitioners and clinical nurse specialists, with expert practice skills can provide high-quality care and solve complex healthcare problems. In general, nurse practitioners are generalist nurses who work as autonomous clinicians with whole personal care. Clinical nurse specialists, in contrast, are nurses with advanced nursing knowledge and skills for individuals or specific populations. Their roles are independent and different; however, similarities exist in their role in potentially improving healthcare outcomes. Although two previous studies described the role of nephrology nurse practitioners, they were systematic reviews, and their outcomes were limited. To clarify the overall aspect of advanced practice nurses’ role, it is necessary to extract the studies illustrating advanced practice nurses’ practices for patients with chronic kidney disease.

**Objective:**

This study aims to map the literature describing the role of advanced practice nurses in improving healthcare outcomes for patients with chronic kidney disease.

**Materials and methods:**

This scoping review will be conducted using the Joanna Briggs Institute methodology for scoping review. Online databases will be searched across MEDLINE (PubMed), CINAHL (EBSCOhost), Cochrane Database of Systematic Reviews, Cochrane Central Register of Controlled Trials, and Web of Science. Only studies published in English will be included, and no date limit will be set. Chronic kidney disease, renal replacement therapy, and advanced practice nurses as keywords and related search terms will be used. Two independent reviewers will screen the title and abstract/full-text; in case of discrepancy, a third reviewer will make the final decision. The results will be extracted and presented following the review question concerning the study characteristics, patients’ characteristics, condition of chronic kidney disease, and role of advanced practice nurses.

## Introduction

The prevalence of all stages of chronic kidney disease (CKD) in the general population is 9–15% [1,2]. According to the report of the Global Burden of Disease, the global prevalence of CKD at all ages has increased by 29.3% (95% UI 26.4 to 32.6) from 1990 to 2017 [1]. CKD is a progressive chronic disease; if patients have multiple comorbidities that progress to renal dysfunction, the status of the kidney progresses more rapidly [3,4]. When the kidneys function inefficiently, waste products and extra fluid accumulate in the body, resulting in a myriad of health problems, including cardiac disease and hypertension [5]. The incidence rate of treated end-stage renal disease (ESRD) is relatively stable, has decreased or increased slightly in some high-income countries, but has increased substantially in others, with a global trend [6]. ESRD negatively affects patients’ quality of life [7,8], leading to increased hospitalization and mortality [9]. It is important that patients with CKD, which is a chronic disease, maintain their daily life and quality of life while controlling complications and receiving necessary treatment; however, to accomplish these tasks, high-quality care is required.

Advanced practice nurses (APNs) are expected to provide a high level of care and be able to cope with the complexity of the problem. APNs, such as nurse practitioners (NPs) and clinical nurse specialists (CNSs), are the most common. The International Council of Nursing describes APNs as “a generalist or specialized nurse who has acquired, through additional graduate education (at least a master’s degree), the expert knowledge base, complex decision-making skills, and clinical competencies for advanced nursing practice [10]. NPs have provided primary, acute, and specialty healthcare to a diverse group of patients of all ages for nearly half a century [11]. The NP education program was established at the University of Colorado in 1965 to increase the primary care workforce and integrate the content into a nursing master’s program [12]. Conversely, the pioneer CNS program was developed in 1956 by Hildegard Peplau in the United States [13–15]. CNSs are expert clinicians focused on a specialist area and provide high-quality care with clinical excellence in multiple healthcare settings [10,14]. NPs and CNSs have autonomy and perform different functions, but they share the core of advanced practice competencies and provide high-quality care [16,17]. Both have overlapping roles in the clinical setting and contribute to solving complex patient problems as APNs [18–20]. In recent years, APNs have spread and developed worldwide [21].

In the nephrology setting, APNs have also provided high-level care for patients with CKD. In the United States, the presence of nephrology APNs in dialysis units was first reported in 1976 [22]. In the early 2000s, some literature described the role of nephrology APNs and showed their practice [23–25]. Nowadays, nephrology APNs have contributed to improving the healthcare outcomes of patients with all CKD stages who are not yet on dialysis [26–28], are on dialysis [29,30], or are pre- or post-kidney transplant [31,32]. We believe it is worthwhile to identify and present such cumulative practice of nephrology APNs. This is because nephrology APNs may have the opportunity to access and share knowledge regarding their roles that they may not be aware of. Of these roles, improving healthcare outcomes for patients with CKD is directly linked to promoting their well-being.

We have identified two systematic reviews that have been done on similar topics [33,34]. Xu et al. compared nurse- and physician-led care for patients with CKD. This meta-analysis showed that a nurse-coordinated care model reduced the risks of composite outcomes, including death, the occurrence rate of end-stage renal disease, and doubled serum creatinine [33]. McCrory et al. reported the outcomes of patients with CKD stages 2–4, where NPs were the primary workforce. The results of this review reported that NPs provided care equal to or superior to that of physicians in terms of blood pressure control, low-density lipoprotein, phosphate, and parathormone levels, as well as glycemic control [34]. NPs did do additional roles under the indirect supervision of a nephrologist in these studies. However, there are a few limitations to these studies. First, because the systematic review was intended to integrate previous research and specific conditions with strict eligibility criteria, the role of APNs for patients on peritoneal dialysis and pre- or post- kidney transplant was excluded. Second, only the NP-level nursing care providers were included in the studies. Then, we cannot view the practice of CNS-level nursing. We consider that there are some gaps in these reviews to describe the role of APNs in improving the healthcare outcomes for CKD. As such, we considered a scoping review to be an appropriate method to research this topic, as it deals with a wide range of subjects and allows better identification of gaps in the current literature. In addition, the results of this scoping review will help APNs access the literature describing their role in nephrology.

### Review question

What is the role of APNs that could improve healthcare outcomes for patients with CKD, and what are their outcomes?

## Materials and methods

This study aims to map the literature describing the role of APNs in improving healthcare outcomes of patients with CKD. This scoping review will be conducted by the Joanna Briggs Institute (JBI) methodology for scoping reviews [35]. The eligibility criteria for study selection are described in terms of participants, concept, context (PCC) framework, and type of sources following JBI methods. The study protocol is reported in accordance with Preferred Reporting Items for Systematic Review and Meta-analysis Protocols (PRISMA-P) (S1 Checklist) [36]. In addition, we will use the Preferred Reporting Items for Systematic Reviews and Meta-Analyses extension for Scoping Reviews (PRISMA-ScR) (S2 Checklist) [37,38] to ensure that this scoping review complies with the scoping review guidelines.

### Eligibility criteria

#### Participants

The review will consider all literature that includes patients aged >18 years with all stages of CKD and describe improved healthcare outcomes after receiving some care from APNs. We will include patients with ESRD receiving any type of renal replacement therapy and include studies of conservative therapy for ESRD, if available. Renal transplantation therapy will be also included in this scoping review.

APNs are “a generalist or specialized nurse who has acquired, through additional graduate education (at least a master’s degree), the expert knowledge base, complex decision-making skills, and clinical competencies for advanced nursing practice.” Globally, the most common APNs are NPs and CNSs [10]. The title of APNs, including NPs/CNSs, differs from each country: advanced practice registered nurse, specialist nurse, nurse clinician, etc [18,21]. Additionally, the definition of APNs varies from country to country, but generally, the education is at the master’s level or higher [18,21]. In this study, we used education at the master’s level or higher as the basis for APNs. For these reasons, the definition of APNs in this study emphasizes concepts rather than titles.

The review will include literature wherein patients received care from APNs in collaboration with specialists in other disciplines.

#### Concept

Our review will provide an overview of the role of nephrology APNs in improving healthcare outcomes for CKD. The role of APNs includes direct care and indirect care; ordering, performing, supervising, and interpreting diagnostic and laboratory tests; making diagnoses; initiating and managing treatment, including prescribing medication and non-pharmacologic treatments; coordinating care; counseling; and educating patients, but not limited to [10,11]. It could also include demonstrating leadership and establishing health care services [10]. We assume that some of the studies that will be screened in our review will not have improved healthcare outcomes significantly. However, it is considered the studies that have not improved healthcare outcomes also have very important findings for APNs. Therefore, we will exclude these studies but will summarize them as appendixes.

#### Context

The review will consider studies conducted in any facility and country. We will consider studies conducted in institutional and non-institutional settings, including hospitals, public or private community clinics, long-term care facilities, and home care.

### Types of sources

This scoping review will consider experimental and quasi-experimental study designs, including randomized controlled trials, non-randomized controlled trials, before and after studies, and interrupted time-series studies. Additionally, analytical observational studies, including prospective and retrospective cohort, case-control, and analytical cross-sectional studies, will be considered for inclusion. Finally, this review will consider descriptive observational study designs, including case series, individual case reports, and descriptive cross-sectional studies but not limited to. Qualitative studies will also be considered if they focus on data including, but not limited to, designs, such as phenomenology, grounded theory, ethnography, qualitative description, and action research. Systematic reviews, meta-analysis, and trial protocols will be excluded. However, references cited in systematic reviews will be deemed suitable for inclusion.

Gray literature and unpublished studies will also be included. However, text and opinion papers will not be considered for inclusion in this scoping review.

### Search strategy

To provide a comprehensive and up-to-date search, a preliminary search of existing systematic reviews about this topic was conducted using the MEDLINE, CINAHL, and Cochrane Database of Systematic Reviews databases on February 20, 2024.

The search strategy will aim to locate both published and unpublished studies. A three-step search will be utilized for each component of this review [35].

An initial limited search of MEDLINE (via PubMed) and CINAHL (via EBSCOhost) will be performed to identify articles on the topic. Thereafter, the words in the titles and abstracts of the relevant articles and the index terms used to describe the articles will be used to develop a complete search strategy. This search strategy will be adapted for each information source, including all the identified keywords and index terms.

A second search using all identified keywords and index terms will be performed across all included databases. An example of a search strategy conducted by MEDLINE (via PubMed) on February 26, 2024, is shown in S1 Table. The databases to be searched will include MEDLINE (via PubMed) and CINAHL (via EBSCOhost), Cochrane Database of Systematic Reviews, Cochrane Central Register of Controlled Trials (CENTRAL; via EBSCOhost), and Web of Science. Databases of gray literature include ProQuest and the first 300 hits on Google Scholar because our initial search results indicate that approximately 300 papers will be suitable for the present study [39]. We will deal with unpublished studies as well; ongoing studies or completed but unpublished studies. The Cochrane Handbook for Systematic Reviews of Interventions recommends using the Trial Registry database as a search strategy for these unpublished studies. Therefore, ClinicalTrials gov. and the International Clinical Trials Registry Platform will be used as our search strategy for unpublished studies.

Third, the reference lists of all the identified reports and articles selected for this scoping review will be screened for additional studies.

Only articles published in English will be included because of the lack of translation services.

When noting missing data or poorly reported items, we aim to contact the author by e-mail, which is considered the most efficient tool [40,41], upon checking the contact information listed in the eligible study or most recent study. Depending on the case, we may consider contacting the authors’ affiliation. We will continue to contact the authors until the completion of the analysis of our scoping review [40,41].

### Study/Source of evidence selection

Following the search, all identified citations will be collated and uploaded into Mendeley V1.19.8 (Mendeley Ltd., Elsevier, Netherlands), and duplicates will be removed. Following a pilot test, titles and abstracts will be screened by two or more independent reviewers to assess whether they meet the inclusion criteria for the review. Potentially relevant sources will be retrieved, and their citation details will be imported into the JBI System for the Unified Management, Assessment, and Review of Information (JBI SUMARI; JBI, Adelaide, Australia). The full texts of the selected citations will be assessed in detail against the inclusion criteria by two independent reviewers. This scoping review will record and report reasons for excluding sources of evidence that do not meet the inclusion criteria in the full text. Any disagreements that arise between the reviewers at each stage of the selection process will be resolved through discussion; if there is a difference in opinion, the decision of a third reviewer will be final.

The search results and study inclusion process will be reported in full in the final scoping review and presented in a PRISMA-ScR flow diagram [37].

### Data extraction

Data will be extracted from the papers included in the review using a charting table aligned by two independent reviewers to the objective and question of this research S2 Table, as indicated by the methodology for scoping reviews developed by JBI [35]. We will extract data about; study characteristics (author, publication year, country, study design, main result), patients’ characteristics (sample size, age, location of patient), condition of CKD (CKD stages of patient, Renal Replacement Therapy type), the role of APNs (title of APNs, types of healthcare provider, characteristics of APNs roles. The draft data extraction tool will be modified and revised as necessary while extracting data from the included sources. The modifications are detailed in this scoping review. Any reviewer disagreements will be resolved through discussion or by a third reviewer.

### Data analysis and presentation

The overview of the reviewed material, where possible and appropriate, will be presented in tabular form along with a narrative summary that aligns with the objectives and scope of this review. A descriptive summary will accompany the tabulated and charted results and describe how the results relate to the review’s objectives and questions. A summary of each article will include the author(s) name, year of publication, country of origin, population, sample size, methodology, concepts of interest, and key findings related to the scoping review question.

## Discussion

CKD is a common chronic disease, and healthcare providers must provide appropriate care. It is expected that advanced practice nursing will solve complex matters for CKD. APNs have provided high-quality care and accumulated evidence by researching and reporting their own practices. We suggest extracting the literature on the role of nephrology APNs in improving healthcare outcomes for all stages of CKD. The results of this scoping review will help nephrology APNs develop their role and identify new gaps in unreported areas.

There is no common understanding of the role of APNs because their scope of practice differs in different countries [42–44]. It is difficult to synthesize these differences in this scoping review. However, we believe that to map this heterogeneous literature, a scoping review is the best method. The methods used in this scoping review protocol are based on the JBI methodology for scoping reviews. In addition, PRISMA-P and PRISMA-ScR were used to ensure that this Scoping review is in accordance with these guidelines. We consider this a methodological limitation because the search strategy may not fully cover all publications and is limited by language and databases. Additionally, the scope of APNs practice in each country is different; hence, the results we are trying to show in this study may not be directly applicable.

## Supporting information

S1 Checklist PRISMA-P 2015 checklist. (DOCX)

S2 Checklist PRISMA-ScR 2018 checklist. (DOCX)

S1 Table Search strategies. (DOCX)

S2 Table Data extraction. (DOCX)
