# Supplementary material for: The role of advanced practice nurses in improving healthcare outcomes for patients with chronic kidney disease: A scoping review protocol
Source: PLoS One. 2024 Apr 4;19(4):e0301676. doi: 10.1371/journal.pone.0301676 (PMC10994302; doi:10.1371/journal.pone.0301676)
Supplement: S1 Table — (DOCX) [file pone.0301676.s003.docx]

Table S3. Search strategies
